# *Pseudomonas aeruginosa* Pore-Forming Exolysin and Type IV Pili Cooperate To Induce Host Cell Lysis

**DOI:** 10.1128/mBio.02250-16

**Published:** 2017-01-24

**Authors:** Pauline Basso, Michel Ragno, Sylvie Elsen, Emeline Reboud, Guillaume Golovkine, Stephanie Bouillot, Philippe Huber, Stephen Lory, Eric Faudry, Ina Attrée

**Affiliations:** a University of Grenoble Alpes, Grenoble, France; b CNRS, ERL5261, Grenoble, France; c CEA, BIG-BCI, Grenoble, France; d INSERM, U1036, Grenoble, France; e Department of Microbiology and Immunobiology, Harvard Medical School, Boston, Massachusetts, USA; University of Washington

## Abstract

Clinical strains of *Pseudomonas aeruginosa* lacking the type III secretion system genes employ a toxin, exolysin (ExlA), for host cell membrane disruption. Here, we demonstrated that ExlA export requires a predicted outer membrane protein, ExlB, showing that ExlA and ExlB define a new active two-partner secretion (TPS) system of *P. aeruginosa*. In addition to the TPS signals, ExlA harbors several distinct domains, which include one hemagglutinin domain, five arginine-glycine-aspartic acid (RGD) motifs, and a C-terminal region lacking any identifiable sequence motifs. However, this C-terminal region is important for the toxic activity, since its deletion abolishes host cell lysis. Using lipid vesicles and eukaryotic cells, including red blood cells, we demonstrated that ExlA has a pore-forming activity which precedes cell membrane disruption of nucleated cells. Finally, we developed a high-throughput cell-based live-dead assay and used it to screen a transposon mutant library of an ExlA-producing *P. aeruginosa* clinical strain for bacterial factors required for ExlA-mediated toxicity. The screen resulted in the identification of proteins involved in the formation of type IV pili as being required for ExlA to exert its cytotoxic activity by promoting close contact between bacteria and the host cell. These findings represent the first example of cooperation between a pore-forming toxin of the TPS family and surface appendages in host cell intoxication.

## IMPORTANCE

The course and outcome of acute, toxigenic infections by *Pseudomonas aeruginosa* clinical isolates rely on the deployment of one of two virulence strategies: delivery of effectors by the well-known type III secretion system or the cytolytic activity of the recently identified two-partner secreted toxin, exolysin. Here, we characterize several features of the mammalian cell intoxication process mediated by exolysin. We found that exolysin requires the outer membrane protein ExlB for export into extracellular medium. Using *in vitro* recombinant protein and *ex vivo* assays, we demonstrated a pore-forming activity of exolysin. A cellular cytotoxicity screen of a transposon mutant library, made in an exolysin-producing clinical strain, identified type IV pili as bacterial appendages required for exolysin toxic function. This work deciphers molecular mechanisms underlying the activity of novel virulence factors used by *P. aeruginosa* clinical strains lacking the type III secretion system, including a requirement for the toxin-producing bacteria to be attached to the targeted cell to induce cytolysis, and defines new targets for developing antivirulence strategies.

## INTRODUCTION

*Pseudomonas aeruginosa* is a major human opportunistic pathogen, frequently associated with nosocomial infections, notably in intensive care units, where it is responsible for approximately 40% of deaths of patients with ventilator-associated pneumonia (1). This Gram-negative bacterium also causes life-threatening chronic infections in cystic fibrosis patients and numerous acute infections of eyes, ears, urinary tracts, and injuries such as burns (2). A combination of several factors, including its metabolic adaptability, a panoply of virulence factors, and the development of multiresistance among clinical strains, makes *P. aeruginosa* extremely difficult to eradicate. Facing the rise in bacterial antibiotic resistance, there is an alarming deficiency in therapeutic options for the majority of human and animal bacterial pathogens. *P. aeruginosa* belongs to the family of so-called “ESKAPE” pathogens (*Enterococcus faecium*, *Staphylococcus aureus*, *Klebsiella pneumoniae*, *Acinetobacter baumannii*, *P. aeruginosa*, and *Enterobacter* species), which effectively “escape” the effects of currently available antibiotics and are considered by the Infectious Diseases Society of America (IDSA) as being the priority pathogens for the urgent development of novel antimicrobial agents (3). One of the strategies for novel therapeutics against bacterial pathogens is based on the discovery of molecules capable of interfering with their determinants of pathogenicity. This “antivirulence” approach requires a complete knowledge of factors and molecular mechanisms used by pathogens for successful colonization of humans and avoidance of host defenses.

Several hundred available whole-genome sequences of various *P. aeruginosa* strains (http://www.pseudomonas.com [4]) were used in phylogenetic analyses to define two distant clades. One of these contains strains previously referred to as “clonal outliers” (5–7); the first identified member of this group is strain PA7 (8). Although the virulence of *P. aeruginosa* is multifactorial, the two clades differ mainly in the way that they exert their cytolytic activity on human cells (6, 9, 10). The genomes of the PAO1/PA14 group contain the determinants of a well-studied export nanomachine called the type III secretion system (T3SS) that allows the injection of four main *P. aeruginosa* effectors harboring enzymatic activities (ExoS, ExoT, ExoY, and ExoU) directly into the host cell cytoplasm (reviewed in reference 11). The virulence of clinical strains from this clade in human infections has been clearly correlated with the synthesis of T3SS and the injection of the four exoenzymes (12–15). The second clade not only lacks the entire T3SS-encoding locus composed of five operons but also lacks the genes encoding the type III secreted exoenzymes (6, 9). We recently discovered a completely novel virulence mechanism used by those strains lacking T3SS (i.e., the PA7-like clade), by studying the highly virulent and cytolytic strain CLJ1, isolated from a patient with hemorrhagic pneumonia. Using quantitative comparative proteomic analysis of CLJ1 secreted proteins, we identified a toxin responsible for eukaryotic cell lysis and named it exolysin (ExlA) (6). Bioinformatics analysis of ExlA showed several sequence features found in two-partner secretion (TPS) systems (16), including an N-terminal general Sec (*sec*retion) export signal peptide followed by a sequence with a TPS motif. Moreover, immediately upstream of *exlA* we identified a gene encoding the putative TPS outer membrane protein and designated it ExlB. It is noteworthy that the *exlBA* genes are present not only in strains of the PA7 clade but also in some clinical and environmental isolates phylogenetically closer to PAO1/PA14-like classical strains (reviewed in reference 9), and their localization between genes corresponding to *PA0874* and *PA0873* is conserved. All *exlBA*^+^ strains characterized until now lack the T3SS locus. Interestingly, the quantity of secreted ExlA differs greatly between the *exlA-*positive strains, determining the degree of their cytotoxic activity and virulence in mice (17).

In this work, we further characterize exolysin secretion and activity *in vivo* and *in vitro*. Using cellular cytotoxicity assays, hemoglobin release, and a liposome leakage assay with purified recombinant proteins, we show that exolysin is a TPS pore-forming toxin (PFT) and that its activity requires its C-terminal domain. A cellular screen of a transposon (Tn) mutant library for the loss of exolysin-dependent cytolysis, created in the ExlA-producing *P. aeruginosa* urinary isolate IHMA879472 (IHMA), resulted in the identification of type IV pili (T4P) as being required for host cell intoxication. This shows that type IV pili are cooperating with ExlA in its cytotoxic activity, probably by mediating bacterial adhesion and establishing adequate ExlA local concentrations.

## RESULTS

### The POTRA domains of the outer membrane partner ExlB are required for ExlA secretion.

**FIG 1:**
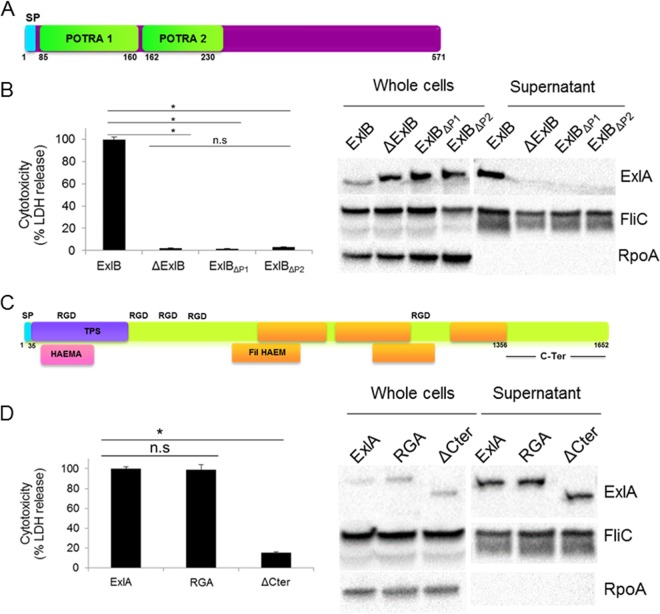
ExlB and ExlA represent a *P. aeruginosa* TPS system, and the ExlA C-terminal region is required for toxicity. (A) ExlB possesses an N-terminal type I secretion signal peptide (SP) and two POTRA domains. (B) (Left) The cytotoxic effect of *P. aeruginosa* PAO1 expressing ExlB mutant proteins on epithelial A549 cells. Analysis of variance was used to compare mutants (*, *P* ≤ 0.05; n.s., not significant). Monolayers were infected with PAO1Δ*xcpR*Δ*pscC*::pSW196*exlBA* (ExlB) or PAO1Δ*xcpR*Δ*pscC*::pSW196*exlA* carrying the deletion of *exlB* (ΔExlB) or the deletion of either POTRA domain 1 (PAO1Δ*xcpR*Δ*pscC*::pSW196*exlB*ΔP1-*exlA*) (ΔP1) or POTRA domain 2 (PAO1Δ*xcpR*Δ*pscC*::pSW196*exlB*ΔP2-*exlA*) (ΔP2), and cytotoxicity was measured by the release of lactate dehydrogenase (LDH). (Right) Immunoblot analysis of the corresponding strains. Western blot assays were performed on bacterial lysates (whole cells) and secreted proteins (supernatant), and proteins were revealed by anti-ExlA antibodies. FliC and RpoA were used as loading controls, probed with the corresponding antibodies. (C) ExlA belongs to the family of polymorphic toxins and carries the N-terminal type I secretion signal peptide (SP), the conserved TPS secretion domain, hemagglutinin-like domains, FHA repeats, and a C-terminal domain of unknown function. ExlA also possesses five RGD motifs. (D) Cytotoxicity assays of A549 cells infected with *P. aeruginosa* PAO1 expressing ExlA mutants (left) (analysis of variance was used to compare mutants [*, *P* ≤ 0.05; n.s., not significant]) and Western blot analysis (right) of PAO1Δ*xcpR*Δ*pscC*::pSW196*exlBA* (ExlA) and penta-RGA PAO1Δ*xcpR*Δ*pscC*::pSW196*exlBA*_RGA_ (RGA) and PAO1Δ*xcpR*Δ*pscC*::pSW196*exlBA*_ΔCter_ (ΔCter) mutants, performed as described for panel B.

We previously showed that neither ExlB nor ExlA alone is able to induce eukaryotic cell lysis (6), suggesting that they form an ExlB-ExlA TPS system. Indeed, only a *P. aeruginosa* strain harboring both *exlB*-*exlA* genes, expressed from an arabinose-inducible promoter, was cytolytic on epithelial and endothelial cells (6). As with other TPS systems, the TpsB homologue ExlB, in addition to the β-barrel-forming domain, possesses two predicted conserved domains predicted to be oriented toward the bacterial periplasm and is responsible for TpsA (ExlA) export (18, 19). These domains are called the POTRA domains (for *po*lypeptide-associated *tra*nsport) and are located in ExlB between amino acids 85 to 160 and 162 to 230 for POTRA1 and POTRA2, respectively (Fig. 1A). In TPS systems, these domains interact with the secreted proteins. In *Bordetella pertussis* TPS filamentous hemagglutinin (FHA)/FhaC, the deletion of the POTRA domains in FhaC does not affect its stability and outer membrane location but completely abolishes the secretion of FHA (20). In order to further demonstrate the dependence of ExlA secretion on ExlB and to assess the role of the two POTRA domains, we deleted the whole *exlB* gene and created two *exlB* mutants in which each POTRA domain is deleted. The mutations were done in the engineered heterologous PAO1 strain lacking both the essential components of the T2SS (Δ*xcpR*) and T3SS (Δ*pscC*), in order to abolish any cytotoxic activity due to these two systems, and harboring *exlB*-*exlA* genes on the integrative plasmid described previously (6). All strains synthesized the ExlA protein, but strains lacking ExlB and either POTRA1 or POTRA2 were unable to secrete it into bacterial supernatants, as visualized by immunoblotting using anti-ExlA antibodies (Fig. 1B). Consequently, the strains lacking ExlB or expressing ExlB without both POTRA domains were noncytotoxic (Fig. 1B). Therefore, we concluded that ExlB is indeed the cognate partner of ExlA permitting its secretion, probably through the interaction between the POTRA domains and the N-terminal TPS signal of ExlA, as previously shown for the FHA and FhaC of *B. pertussis* (21–23).

### The C-terminal part of ExlA, but not the five RGD motifs, confers on the protein its cytolytic activity.

ExlA is a 172-kDa protein belonging to the family of polymorphic toxins (24). In addition to the conserved secretion motifs based on an analysis in the Pfam database (http://pfam.xfam.org), ExlA shares five β-sheet domains (Fil HAEM) and one hemagglutinin domain (HAEMA) (Fig. 1C) with FHA (25). The ExlA protein also carries five arginine-glycine-aspartic acid (RGD) motifs that are often involved in cell-to-cell recognition with proteins of the integrin family (26). As well, in the majority of polymorphic toxins, as well as in the filamentous hemagglutinin adhesion protein FHA (27), the C-terminal portions specify various activity functions and share conserved features (24), However, the ExlA C-terminal part of approximately 300 amino acids shares no homology with any other protein or domain of known function. Therefore, we undertook to determine the roles of the RGD motifs and the C-terminal domain in cytotoxicity. To that aim, in ExlA we replaced the five RGD motifs with RGA, a mutation known to prevent integrin binding, and also engineered a truncated ExlA (ExlA_ΔCter_) which lacks the last 296 amino acids. Both mutant proteins were produced and secreted at the same level as the wild-type protein (Fig. 1D). Cytotoxicity assays on epithelial A549 cells showed that while the ExlA_RGA_ pentamutant exhibited high cytotoxicity (90%) similar to that of the wild-type ExlA, ExlA_ΔCter_ completely lost the ability to lyse epithelial cells (Fig. 1D), showing that the cytolytic activity of the ExlA protein requires its C-terminal domain but does not depend on the RGD motifs.

### ExlA forms pores in RBCs.

**FIG 2:**
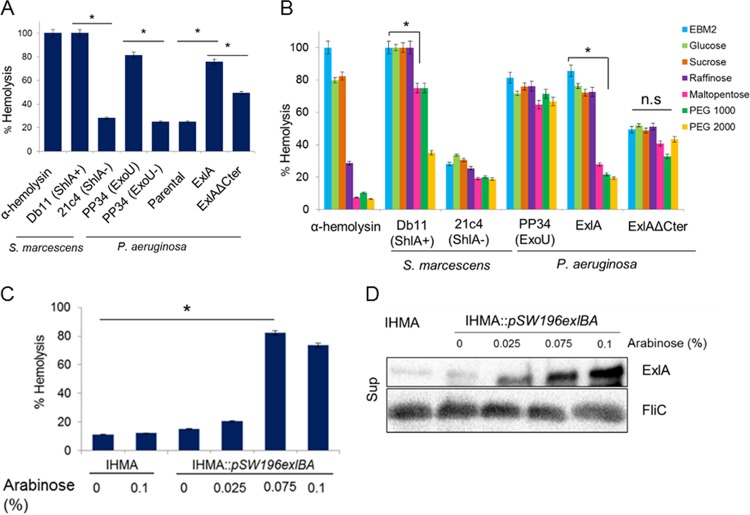
Exolysin is a pore-forming toxin. (A) Lysis of RBCs incubated with α-hemolysin from *S. aureus* or infected (at an MOI of 10) with *S. marcescens* secreting ShlA (Db11), an *S. marcescens* ShlA^*−*^ mutant (21c4), *P. aeruginosa* strain PP34 expressing T3S toxin ExoU (ExoU), *P. aeruginosa* PP34ΔExoU, the *P. aeruginosa* PAO1Δ*xcpR*Δ*pscC* strain (Parental), and the *P. aeruginosa* PAO1Δ*xcpR*Δ*pscC*::*pSW196exlBA* strain secreting ExlA (ExlA) or the *P. aeruginosa* PAO1Δ*xcpR*Δ*pscC*::*pSW196exlBA*_ΔCter_ strain secreting ExlA_ΔCter_ (ExlAΔCter). (B) Hemolysis assays performed with α-hemolysin (100 nM) and the indicated strains as described in the legend to Fig. 3A in the presence of 30 mM sugars and PEG osmoprotectants. (C) Hemolysis following incubation with *P. aeruginosa* IHMA or IHMA::*pSW196exlBA*. For induction of *exlBA*, bacteria were grown in the presence of 0.025%, 0.075%, and 0.1% arabinose for 3 h and used at an MOI of 10. In a control, the hemolysis assay was done with IHMA in the presence of arabinose (0.1%) in EBM-2. In panels A to C, hemolysis was determined by measuring the release of hemoglobin into the supernatants (OD_549_). Complete (100%) lysis of RBCs was accomplished using 1% SDS. Statistical analysis was performed by analysis of variance (*, *P* ≤ 0.05; n.s., not significant). (D) Western blot analysis of ExlA secreted by IHMA or IHMA::*pSW196exlBA* in the presence of arabinose. FliC (Sup) probed with anti-FliC antibody was used as a loading control.

Strains expressing ExlA induce plasma membrane rupture of cultured eukaryotic cells, leading to rapid cell death (within 3 h postinfection) (6). To further decipher the mechanism of ExlA action, we examined its capacity to form pores within membranes, as is the case for several bacterial membrane-damaging toxins, including ShlA of *Serratia marcescens* (28). The classical model to study the ability of a toxin to form pores in biological membranes utilizes red blood cells (RBCs), where pore formation is evaluated by measuring hemoglobin release (29). RBCs were incubated with *P. aeruginosa* strains expressing different ExlA variants, and hemoglobin release was measured after 90 min of coincubation. The well-characterized pore-forming toxin α-hemolysin from *Staphylococcus aureus*, *S. marcescens* expressing ShlA, and *P. aeruginosa* PP34, which produces the T3SS-exported phospholipase ExoU, all induced more than 90% hemolysis within 90 min of incubation (Fig. 2A). Negative controls included *S. marcescens* strain 21c4, unable to secrete ShlA, and *P. aeruginosa* lacking ExoU (PP34Δ*exoU*), which showed weak (ca. 20%) hemolysis (Fig. 2A). The T2SS/T3SS-deficient *P. aeruginosa* PAO1*ΔxcpRΔpscC* (“Parental” in Fig. 2A) was poorly hemolytic with approximately 25% hemolysis, whereas expression of *exlB*-*exlA* in the same strain resulted in >80% hemolysis. As already observed for the cytolysis of A549 cells, *P. aeruginosa* expressing ExlA_ΔCter_ induced reduced hemolysis; however, this was higher (by about 50%) than that of the parental ExlA^−^ strain (Fig. 2A). For the demonstration that ExlA is indeed a pore-forming toxin, we approximated the pore size using a variety of osmoprotectants in the cytolytic assays. In these experiments, osmoprotectants (sugars and polyethylene glycols [PEGs]) of different sizes allowed us to evaluate the diameter of the pore (Fig. 2B). As expected based on a previous report (30), the α-hemolysin forms a pore of about 1 nm and the hemolysis is inhibited by raffinose, while ShlA forms a pore of about 2 nm, with hemolysis being inhibited by PEG 2000 (31). Hemolysis induced by ExoU was not inhibited by any sugars or PEGs, an observation in agreement with the fact that ExoU is not a pore-forming toxin but a potent phospholipase (32). The smallest molecule inhibiting ExlA-induced hemolysis was maltopentaose, which indicated that the diameter of the ExlA pore is approximately 1.6 nm, based on the hydrodynamic radius of the osmoprotectant molecules, as described previously (29). In contrast, the 50% hemolysis generated by ExlA_ΔCter_ could not be inhibited by any of the osmoprotectants. This response is similar to that observed with ExoU, suggesting that this hemolysis was not due to pore formation but rather was caused by some other membrane-disruptive activity detectable on RBCs. Initially, we observed that natural clinical isolates synthetizing ExlA are in general poorly hemolytic (9). We hypothesized that the difference between the clinical isolates and the PAO1 strain ectopically overexpressing ExlA, which was used in the hemolytic assays, is due to the difference in the quantities of ExlA secreted by individual strains. Since PA7 is not amenable to genetic manipulation, we selected strain IHMA, a clinical isolate from our laboratory collection that secretes ExlA and is lytic on epithelial cells (see Table S1 in the supplemental material). In this strain, we introduced pBAD-*exlB*-*exlA* into the chromosome and performed a hemolysis assay using variable arabinose concentrations to induce different levels of *exlA* expression. Indeed, raising the concentrations of arabinose significantly increased the capacity of a clinical strain to lyse RBCs in a dose-dependent manner (Fig. 2C), and this lytic activity correlates with the increased quantity of secreted ExlA (Fig. 2D).

10.1128/mBio.02250-16.8 TABLE S1  List of 20 Tn mutants selected after secondary screen displaying reduced cytotoxicity (<30%) compared to the parental strain IHMA. Download TABLE S1, DOCX file, 0.02 MB. Copyright © 2017 Basso et al. 2017 Basso et al. This content is distributed under the terms of the Creative Commons Attribution 4.0 International license.

### Both the recombinant ExlA and its C-terminal domain induce liposome leakage.

**FIG 3:**
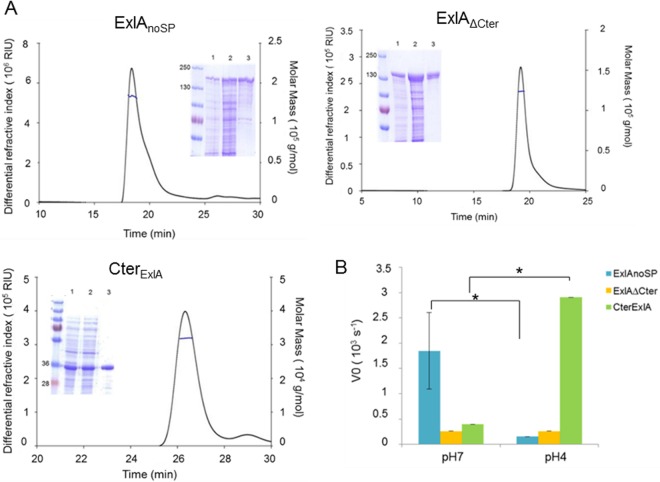
ExlA induces dye leakage from PC/PS liposome. (A) SEC-MALLS analysis of three ExlA variants, ExlA lacking the signal peptide (ExlA_noSP_), ExlA_noSP_ lacking the C-terminal domain (ExlA_ΔCter_), and the C-terminal fragment of ExlA (Cter_ExlA_), showing their soluble and monomeric form. The insets show SDS-PAGE analyses of the corresponding proteins at different stages of purification (lane 1, total lysate; lane 2, soluble lysate; lane 3, eluted fraction after the second step of purification). The values to the left are molecular masses in kilodaltons. (B) Permeabilization of PC/PS liposomes by ExlA proteins. Sulforhodamine B-containing liposomes composed of PC and PS were incubated with ExlA proteins, and the release of SRB was measured over 500 s. The initial rate of dye release (*V*0) was plotted for the three ExlA proteins, at pH 4 and pH 7. Statistical analysis was done by analysis of variance (*, *P* ≤ 0.05).

The observed phenotypes of ExlA-expressing *P. aeruginosa* strains on nucleated cells and on RBCs strongly suggest that ExlA may directly interact with membranes to induce pore formation. To further gain insight into the mode of action and to evaluate the association of the protein with membranes, we purified recombinant ExlA variants and tested their ability to induce membrane damage on the phospholipid bilayers of lipid vesicles. Three ExlA variants were designed for the experiment: (i) the full-length ExlA protein lacking the N-terminal export signal peptide and starting at Gly^35^ (ExlA_noSP_), (ii) a truncated version of ExlA lacking the last C-terminal 296 amino acids (ExlA_ΔCter_), and (iii) a polypeptide comprising the C-terminal 296-amino-acid region (Cter_ExlA_). After optimization of the overexpression conditions and purification steps (see Materials and Methods for further details), the three variants were obtained in soluble forms and in quantities required for biochemical characterization (1, 6, and 28 mg of protein/liter of culture for ExlA_noSP_, ExlA_ΔCter_, and Cter_ExlA_, respectively). Proteins were stable except for ExlA_noSP_, which over time showed some degradation (Fig. 3A). Size exclusion chromatography with detection by multiangle laser light scattering analysis (SEC-MALLS) of the three variants showed that all of them are produced as soluble monomers (Fig. 3A). The various proteins were tested in a liposome leakage assay as described previously (33, 34, 35) (Materials and Methods), where the large unilamellar vesicles (LUVs) incorporating sulforhodamine B (SRB) were prepared by freeze-thaw cycles followed by extrusion and subsequently checked by dynamic light scattering. As a control for vesicle preparation, we used the T3SS translocator PopB, which was previously shown to promote membrane permeabilization at pH 5 (33, 35). We tested the three ExlA variants at various pHs and on LUVs consisting of phosphatidylcholine (PC) alone or a mixture of PC and phosphatidylserine (PS) or other lipids. ExlA_noSP_ was highly efficient in inducing SRB leakage from LUVs composed of PC/PS at neutral pH (Fig. 3B), whereas other types of LUVs were resistant to permeabilization even at higher protein concentration (up to 5 times the initial concentration) (Fig. S1). For comparison, ShlA similarly requires PS and PC for pore formation, and the addition of phosphatidylethanolamine (PE) furthermore increases the susceptibility of LUVs to ShlA (34). ExlA_noSP_ lacking the C-terminal region (ExlA_ΔCter_) was unable to induce significant liposome leakage at any tested pH, confirming the experiments on nucleated cells, which showed that the C-terminal domain is required for cytolysis (Fig. 3B). Moreover, the 35-kDa polypeptide comprising only the C-terminal domain (Cter_ExlA_) possesses on its own a significant membrane permeabilization activity, though exclusively at pH 4 (Fig. 3B). This result shows that the Cter_ExlA_ protein’s activity requires conformational changes induced by low pH and these are not required for the full-length protein activity, suggesting that the N-terminal domain contributes to conformational changes required for membrane binding and the induction of liposome leakage.

10.1128/mBio.02250-16.3 FIG S1  Kinetics of sulforhodamine B (SRB) release from liposomes after ExlA incubation at pH 7. SRB-containing liposomes composed of PC/PS, PC, PC/PS/PE, or PC/PE were incubated with ExlA protein at pH 7, and the release of SRB was measured over 500 s. Download FIG S1, TIF file, 0.1 MB. Copyright © 2017 Basso et al. 2017 Basso et al. This content is distributed under the terms of the Creative Commons Attribution 4.0 International license.

### Kinetics of pore formation and membrane disruption on nucleated cells seen by video microscopy.

**FIG 4:**
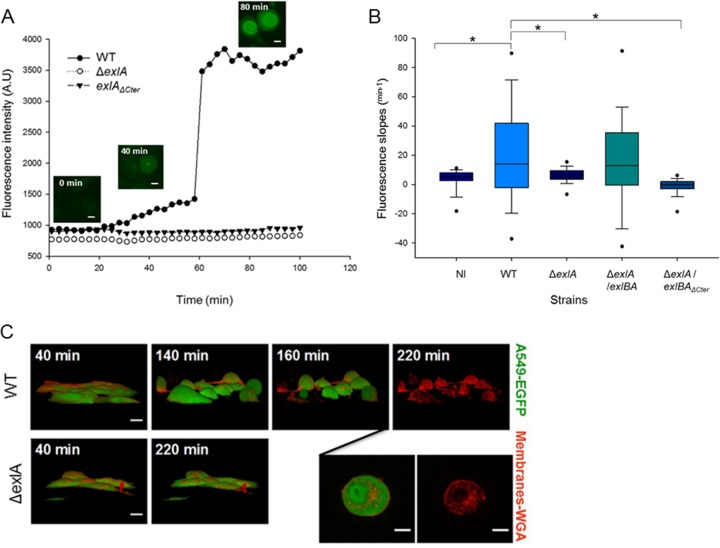
ExlA-induced pore formation precedes plasma membrane disruption. (A) Incorporation of YoPro-1 (green) into infected A549 cells was visualized and measured by time-lapse microscopy. The representative images (inset figures) taken with an ArrayScan wide-field microscope are shown for the wild-type infection. Bars, 10 μm. A549 cells were infected with *P. aeruginosa* IHMA (WT), IHMAΔ*exlA* (*ΔexlA*), and IHMA*exlA*_ΔCter_ (*exlA*_ΔCter_). (B) YoPro-1 incorporation was analyzed for *P. aeruginosa* IHMA (WT)-, IHMAΔ*exlA* (Δ*exlA*)-, IHMAΔ*exlA*::*pSW196exlBA* (Δ*exlA/exlBA*)-, and IHMAΔ*exlA*::*pSW196exlBA*_ΔCter_ (Δ*exlA/exlBA*_ΔCter_)-infected A549 cells and compared to a noninfected (NI) control. Fluorescence intensities were measured using HCS studio analysis software. The box plot represents slopes of phase I YoPro-1 intensities (from 30 to 60 min postinfection) (*n* = 100 cells per condition). Asterisks indicate statistically significant differences (Kruskal-Wallis, *P* < 0.05). (C) A549-EGFP cells, whose plasma membranes were labeled with WGA-Alexa 647, were infected at an MOI of 10 with *P. aeruginosa* IHMA (WT) or IHMAΔ*exlA* (Δ*exlA*) and observed by confocal spinning-disk microscopy. Two inset images represent the same cell infected by the wild-type strain at 200-min and 220-min time points. Note the cell rounding (at 140 min postinfection), absence of EGFP labeling, and rupture of cell membrane (at 220 min postinfection) for infection with the wild-type strain (WT). 3D images were constructed from *z*-planes. Bars, 10 μm. The film with the entire sequence is provided in Movie S1 in the supplemental material.

Having established that ExlA displays a pore-forming activity on RBCs and liposomes, we sought to determine the kinetics of the various events leading to the death of eukaryotic cells induced by clinical strains of *P. aeruginosa* expressing ExlA. To that aim, we observed epithelial A549 cells infected with IHMA or its genetic variants by time-lapse microscopy. To follow the plasma membrane permeability, the infection was performed in the presence of YoPro-1, a membrane-impermeant dye (629 Da) that fluoresces upon interaction with RNA in the cytoplasm and DNA in the nucleus and can be used as a marker of small pores occurring during early apoptosis (Invitrogen). In A549 cells infected with ExlA-expressing strains, we observed and measured an increase of YoPro-1 fluorescence at early time points in the cytoplasm and later in the nucleus (as illustrated in Fig. 4A). The data show an initial phase of slow fluorescence increase from 30 to 60 min corresponding to the complete entry of the dye in the cytoplasm. This is followed by a phase of rapid increase of fluorescence until it reaches a plateau corresponding to the entry into the nucleus. Infection with IHMAΔ*exlA* and IHMA*exlA*_*ΔCter*_ did not show any fluorescence in the cytoplasm or in the nuclei at any time points (Fig. 4A). We quantified the first phase of YoPro-1 incorporation on 100 cells for each condition and found significant differences in calculated slope values for IHMA versus IHMAΔ*exlA* and IHMA versus IHMAΔ*exlA*::pSW196*exlBA*_*ΔCter*_ (Fig. 4B). We conclude that the ExlA-induced pore formation, as visualized by YoPro-1 incorporation, starts at 30 min postinfection. To further characterize early steps of ExlA-induced morphological changes in epithelial cells, we performed time-lapse microscopy with a confocal microscope to generate three-dimensional (3D) images. A549-EGFP (enhanced green fluorescent protein) cells, with membranes labeled with wheat germ agglutinin (WGA)-Alexa 647, were infected with various strains secreting ExlA. Under these conditions, we observed that IHMA-infected cells initially became round (Fig. 4C; time 140 min), followed by a loss of their internal EGFP content, leaving shells of empty plasma membranes (Fig. 4C; time 220 min). Therefore, ExlA rapidly forms pores in the membranes of A549 cells, allowing the entry of small molecules (such as YoPro-1) into the cytoplasm. At later time points, the cell morphology changes and cells become round. ExlA eventually leads to plasma membrane rupture, an event detected by GFP release and visualized by discontinuous WGA labeling.

10.1128/mBio.02250-16.1 MOVIE S1  The A549-EGFP cells labeled with WGA-Alexa 647 were infected with IHMAΔ*exlA* (left) and IHMA (right) at an MOI of 10 and imaged by confocal spinning-disk microscopy for 4 h. The 3D images were constructed from 150 *z*-planes. The film was assembled by ImageJ from 19 time points taken every 20 min. Download MOVIE S1, AVI file, 4.8 MB. Copyright © 2017 Basso et al. 2017 Basso et al. This content is distributed under the terms of the Creative Commons Attribution 4.0 International license.

### Type IV pili are required for ExlA-dependent cytolysis.

**FIG 5:**
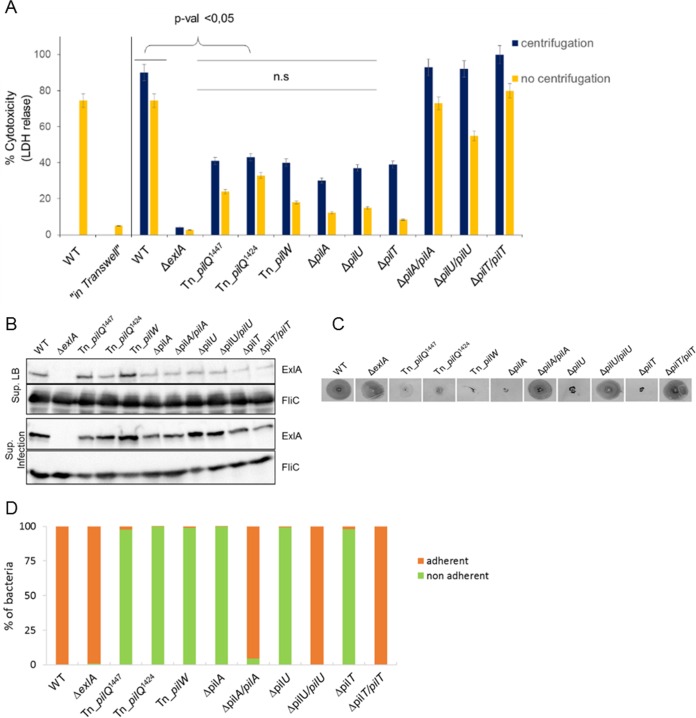
Type IV pili are required for ExlA-dependent cytolysis. (A) Cytotoxicity assays were performed using A549 cells and mutants identified during screening of a transposon (Tn) library, strains with engineered chromosomal deletions (Δ*pilA*, Δ*pilU*, and Δ*pilT*), and the complemented strains. The LDH release was measured as described in the Fig. 1 legend. Where indicated, infections were done in the Transwell system, where bacteria and A549 cells were separated with a membrane. Note the absence of LDH release for “in Transwell” conditions. When indicated, centrifugation was performed immediately after initiation of infection. Analysis of variance was used to compare *pil* mutants to WT (*P* ≤ 0.05; n.s., not significant). (B) Secretion of ExlA in *pil* mutants. Immunoblot analysis was performed using anti-ExlA and anti-FliC antibodies on proteins TCA precipitated from the LB growth medium (Sup. LB) or from cell culture medium following infection of A549 cells with the bacteria (Sup. Infection). (C) Twitching motility of IHMA (WT), IHMAΔ*exlA* and *pil* mutants, and complemented strains, assessed by Coomassie blue staining of motility plates after 48 h. (D) Adhesion of *P. aeruginosa* IHMA, IHMAΔ*exlA* and various *pil* mutants, and complemented strains to A549 cells, quantified after 30 min of infection by enumerating the bacteria (as CFU) in cell-associated (adherent) and supernatant (nonadherent) fractions.

Our preliminary experiments indicated that secreted ExlA, recovered from the medium (LB) used to grow *P. aeruginosa* or from cell culture medium, is unable to induce epithelial cell lysis. Moreover, addition of chloramphenicol to washed bacteria prior to infection abolished the bacterial cytotoxicity, suggesting the requirement for *de novo* ExlA synthesis for efficient pore formation (Fig. S2A). Additionally, recombinant ExlA variants incubated with nucleated cells (epithelial A549 cells and RAW macrophages) and RBCs had no detectable cytolytic activity (Fig. S2A, B, and C). These observations suggest that ExlA is relatively unstable following secretion into the medium and, consequently, its action requires a contact between the toxin-producing bacteria and the eukaryotic cell. To determine whether a contact was indeed important for cytotoxicity, we used a Transwell system in which bacteria and the monolayer of A549 cells are separated by a filter (0.2-µm-diameter pores), permeable by proteins but not the bacteria. Cytotoxicity after “in Transwell” infection was determined by the measurement of lactate dehydrogenase (LDH) release from A549 cells into the supernatant and compared to direct infection conditions (without separation by a filter) performed in parallel (Fig. 5A). When the bacteria are separated from the eukaryotic cells in the Transwell chamber, no LDH release at any time point can be detected and the result is comparable to that seen with an *exlA* mutant. In contrast, infection under normal conditions induced the release of >90% of total cell LDH within 90 min postinfection. Therefore, it appears that the ExlA cytotoxic effect depends on the bacteria being in close proximity to or even in contact with the eukaryotic target cell.

10.1128/mBio.02250-16.4 FIG S2  Cytotoxicity and hemolysis induced by ExlA. (A) Cytotoxicity assay of *P. aeruginosa* expressing different ExlA mutant proteins on A549 cells. Histogram shows the viability of epithelial A549 cells infected with *P. aeruginosa* IHMA (WT) or Δ*exlA* strains, incubated with the supernatant of IHMA culture (“Sup. WT”), 0.5 mg/ml of purified recombinant proteins (ExlA, ExlA_ΔCter_, or Cter_ExlA_), or bacteria washed with LB (“WT Washed LB”) or LB plus chloramphenicol (25 µg/ml) (“WT Washed +Cm”). The cytotoxicity was measured by the release of LDH. (B) Cytotoxicity assay on macrophage cell line RAW 264.7 with 0.5 mg/ml of purified proteins (ExlA, ExlA_ΔCter_, or Cter_ExlA_). (C) Hemolysis with 0.5 mg/ml purified proteins (ExlA, ExlA_ΔCter_, or Cter_ExlA_). The hemolysis assay was performed as described for Fig. 3A. In panels B and C, infections by the wild-type and Δ*exlA* strains were done as positive and negative controls, respectively. Download FIG S2, TIF file, 0.1 MB. Copyright © 2017 Basso et al. 2017 Basso et al. This content is distributed under the terms of the Creative Commons Attribution 4.0 International license.

We thus hypothesized that other bacterial factors, possibly adhesive organelles, may be required for ExlA-dependent cytolysis. To identify these factors, we developed a miniaturized cellular assay to screen a *mariner* transposon (Tn) library of *P. aeruginosa* for the loss of ExlA-mediated cytolysis. The clinical isolate *P. aeruginosa* IHMA was mutagenized by random insertions of the transposon, and a library of 7,400 mutants was obtained. A cytotoxicity test utilizes A549 cells, in which Draq7 and vital Hoechst staining revealed dead and live cells, respectively. Fluorescence images on both channels detecting Draq7 and Hoechst labeling were acquired with an automated high-throughput microscope. The primary screen was carried out in 384-well plates and yielded >200 low-cytotoxicity or noncytotoxic mutants using 30% cytotoxicity as a cutoff. The secondary screen was done manually in 96-well plates by measuring LDH release from infected A549 cells. Finally, 20 mutants were selected as being noncytotoxic, showing similar kinetics and levels of LDH release as the negative control IHMAΔ*exlA*. The transposon insertion sites were determined by semiarbitrary PCR, sequencing, and a BLAST search against the PA7 genome on the *Pseudomonas* website (http://www.pseudomonas.com). Interestingly, out of the 20 selected mutants (Table S1), three mutants harbor the transposon insertion in genes shown previously to be required for type IV pilus biogenesis, with two different Tn insertions within *pilQ* encoding the outer membrane secretin (36) and one insertion in *pilW* encoding a minor pilus subunit (37). These results strongly suggest that T4P are necessary for ExlA to exert its full cytolytic activity. To support this conclusion, we engineered additional isogenic deletions affecting formation of type IV pili, including a mutant lacking the main T4P subunit PilA, and strains with deletions of *pilU* and *pilT* genes, encoding the two ATPases required for pilus extension and retraction (38, 39) (IHMAΔ*pilA*, IHMAΔ*pilU*, and IHMAΔ*pilT*). We also created the corresponding complemented strains (IHMAΔ*pilA*::*pilA*, IHMAΔ*pilU*::*pilU*, and IHMAΔ*pilT*::*pilT*, respectively). We examined these strains for cytotoxicity, ExlA secretion, twitching, and adhesion to eukaryotic cells. In agreement with the results obtained from the transposon library screen and the levels of cytotoxicity provoked by the original transposon T4P mutants, the *pilA*, *pilU*, and *pilT* deletions significantly affected the cytotoxicity, while complemented strains restored the cytotoxicity to wild-type levels (Fig. 5A). Interestingly, bacteria affected in the biogenesis of the pili were still able to secrete ExlA (Fig. 5B), both into LB medium and during epithelial cell infection, suggesting that pili are not required for toxin expression and/or production but rather for promoting its cytolytic activity. We tested also the twitching motility of the mutants and their capacity to adhere to eukaryotic cells. As expected, mutants deleted for *pilA*, *pilU*, or *pilT* were not able to twitch. The complemented strains restored the twitching motility to the same level as IHMA (Fig. 5C). By comparing the CFU of bacteria attached and not attached to cells, we found that the *exlA*-deleted mutant adhered to cells in the same manner as the wild-type strain. All *pil* mutants, including the *pilU* and *pilT* mutants that were reported previously to produce pili but without the capacity to retract, could not adhere to cells (Fig. 5D). To establish whether the defect in pilus biogenesis affected the bacterial cytotoxic behavior and adhesion on different cell lines, the RAW macrophages were infected with different strains. As shown in Fig. S3 and S4, the absence of pili affected the ExlA-dependent cytotoxicity and bacterial adhesion to RAW macrophages to a similar extent as seen with A549 cells. Finally, to determine whether bringing the bacteria lacking pili artificially to the host cell surface could restore their cytotoxicity, we performed a centrifugation step immediately after infection. In all cases, the centrifugation increased the level of cytotoxic activity of pilus mutants, albeit without reaching the wild-type levels (Fig. 5A and S3).

10.1128/mBio.02250-16.5 FIG S3  Cytotoxicity of *P. aeruginosa* mutants on macrophage cell line RAW 264.7. Histogram shows the viability of RAW cells. Cytotoxicity assay of *pil* mutants with or without a centrifugation step. The cytotoxicity was measured by the release of LDH. Analysis of variance was used to compare the *pil* mutant to the WT (*, *P* ≤ 0.05). Download FIG S3, TIF file, 0.1 MB. Copyright © 2017 Basso et al. 2017 Basso et al. This content is distributed under the terms of the Creative Commons Attribution 4.0 International license.

10.1128/mBio.02250-16.6 FIG S4  Adhesion of *P. aeruginosa* to the macrophage cell line RAW 264.7. Adhesion of IHMA, IHMAΔ*exlA* and various *pil* mutants, and complemented strains to macrophages alone (A) and macrophages treated with cytochalasin D (B). Download FIG S4, TIF file, 0.1 MB. Copyright © 2017 Basso et al. 2017 Basso et al. This content is distributed under the terms of the Creative Commons Attribution 4.0 International license.

These experiments demonstrate that adhesion of bacteria to cells is required for ExlA activity. Taken together, these results show that pili are mediating close contact between bacteria and eukaryotic cells, promoting localized action of ExlA, and that this interaction is required for the formation of the pores and subsequent cell death.

## DISCUSSION

In this study, we further deciphered the structure-activity relationship and the biological function of exolysin, the recently identified potent toxin expressed by clinical strains of *P. aeruginosa* lacking T3SS (6, 9, 17). We first confirmed that ExlA requires the accessory protein ExlB for cytotoxic activity on epithelial cells. This finding, together with sequence analysis of the *exlBA* locus, shows that in *P. aeruginosa* ExlB and ExlA form a TPS system (TpsBA), in which *exlB* encodes the outer membrane protein TpsB serving as a cognate porin for the secretion of a TpsA protein, *P. aeruginosa* ExlA. All TpsB proteins are characterized by a domain capable of forming a β-barrel channel in the outer membrane and two periplasmic polypeptide transport-associated (POTRA) domains (18). As for other model TpsB proteins (16), secretion analysis and cytotoxicity assays showed that ExlB is required for ExlA secretion and cytolysis of epithelial cells, demonstrating that ExlB-ExlA constitute a new member of the TPS family.

ExlA is a 172-kDa secreted toxin composed of multiple distinct domains. In the majority of polymorphic proteins of the TpsA family, the activity of the protein resides within their C-terminal regions (24). A C-terminal deletion reduced the ExlA cytolytic activity by 85% without affecting its secretion; this is analogous to the effect of C-terminal deletion in FHA, which abolishes its adhesive properties (27). The secondary structure prediction of the C-terminal domain using the PSIPRED Protein Sequence Analysis Workbench (http://bioinf.cs.ucl.ac.uk/psipred/) and the Phyre2 web portal for protein modeling, prediction, and analysis (http://www.sbg.bio.ic.ac.uk/phyre2) (40) indicates a mixture of β-strands and α-helices (see Fig. S5 in the supplemental material). However, attempts to predict its tertiary structure with Phyre2 did not return any statistically reliable result, as the best model exhibited only 17.5% confidence with a coverage of 19% of the sequence. The N-terminal part of the ExlA protein could adopt an elongated form dominated by β-strands, as is the case for several proteins from the hemagglutinin/hemolysin family, represented by FHA and ShlA. In addition to secretion signals (type I secretion signal peptide and TPS) and hemagglutinin domains, ExlA possesses five RGD motifs and the C-terminal domain sharing no homology with any other proteins. The penta-RGD mutant retained full cytolytic activity, implying that ExlA uses other ways to interact with eukaryotic cell surfaces, at least under our *ex vivo* conditions. RGD motifs have been found in other bacterial adhesion molecules, such as in filamentous hemagglutinin of *Bordetella*; however, their role in cell host interaction could not be clearly demonstrated (41).

10.1128/mBio.02250-16.7 FIG S5  Schematic representation of secondary structure prediction for the C-terminal domain (residues 1356 to 1656) of ExlA. Green arrows and orange cylinders correspond to β-strands and α-helices, respectively. The prediction was done by PsiPred and Phyre2. Download FIG S5, TIF file, 0.1 MB. Copyright © 2017 Basso et al. 2017 Basso et al. This content is distributed under the terms of the Creative Commons Attribution 4.0 International license.

The efficient cell destruction by *P. aeruginosa* ExlA and the similarity with ShlA of *S. marcescens* prompted us to examine the capacity of exolysin to form pores in biological membranes, characteristics of various secreted bacterial toxins. Using two classical models for studying pore-forming toxins (PFTs), we established that ExlA, upon interaction with cell membranes or artificial lipid vesicles, gives rise to pores of 1.6 nm that ultimately result in leakage of hemoglobin from RBCs and release of fluorescent molecules from liposomes. We further show that the C-terminal domain is required for pore-forming activity. On different types of nucleated cells (epithelial/endothelial cells, macrophages, and lymphocytes), ExlA-expressing *P. aeruginosa* strains cause the release of the cytoplasmic enzyme LDH 3 to 4 h postinfection (17). Using 3D time-lapse microscopy and membrane-impermeant fluorescent dyes, we demonstrate that the membrane rupture is preceded by the formation of membrane pores revealed by the uptake of YoPro-1 within the cell cytoplasm. Therefore, ExlA performs clearly different activity on eukaryotic cells than do any T3S effectors, e.g., ExoS, ExoT, and ExoY, which enzymatically modify intracellular signaling molecules, and ExoU, which destroys membranes by its phospholipase activity (reviewed in reference 11).

The majority of PFTs can be grouped into two families, the α- and β-PFTs; they are secreted as soluble, monomeric molecules that undergo conformational change upon membrane binding followed by formation of membrane-embedded multimers (42). ExlA shares no sequence homology with any other PFTs for which the mechanism of action has been extensively studied, and nothing is known about molecular mechanisms involved in pore formation by the closest ExlA homologue, ShlA. Therefore, the mechanisms of membrane binding, the oligomerization state, and the mechanism of pore formation by this toxin could not be assessed at present.

**FIG 6:**
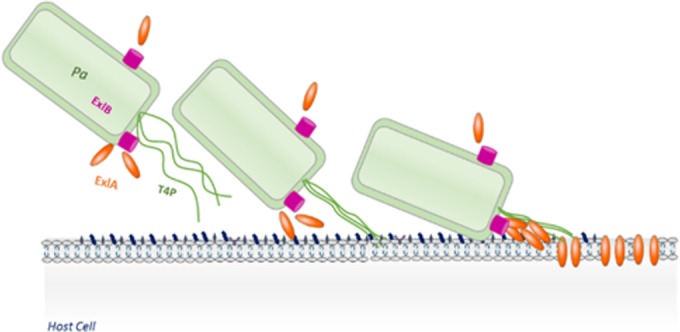
Schematic view of cooperation between T4P and ExlA in cytotoxicity. T4P are required for adhesion of bacteria and promote direct contact between bacterial and mammalian cell surfaces. This brings ExlA-secreting *P. aeruginosa* (*Pa*) to the proximity of the host cell membrane, increasing its local concentration at the site of action. The interaction of ExlA with membranes results in pore formation, followed by LDH release and death of infected eukaryotic cells. ExlB is the cognate outer membrane transporter of ExlA.

Recombinant ExlA, at high concentrations, was able to induce *in vitro* liposome leakage, strongly suggesting that the protein adopts the proper pore conformation following lipid binding. Interestingly, the C-terminal part of the protein, essential for ExlA-mediated *P. aeruginosa* cytolytic activity, was also able to induce liposome leakage, albeit only at acidic pH. This suggests that the first part of the protein, composed of several hemagglutinin domains organized in β-sheets, may play a role in lipid binding and/or protein oligomerization, or conformational changes at the C terminus, accounting for the difference in activity between the whole protein and the C-terminal domain alone. ExlA provokes a liposome leakage only in the presence of charged phospholipids (PS), probably involving electrostatic and hydrophobic interactions; a similar effect is seen for T3SS translocon proteins PopB and PopD (35) and several other membrane-binding proteins, such as colicins (43). In general, PFTs often recognize target cells either through binding to specific lipids or by recognition of a cognate receptor which drastically increases their local concentration at the membrane. This high local concentration promotes their oligomerization and membrane incorporation (42). Neither ExlA harvested from *P. aeruginosa* supernatant nor *in vitro*-purified ExlA variants showed lytic activity on nucleated cells, comparably to what was found for the T3SS toxins (35, 44). Moreover, only bacteria in contact with eukaryotic cells were able to exert ExlA-dependent cytolysis. We postulated that *P. aeruginosa* could have adopted a strategy for ExlA-dependent cytolysis conceptually similar to the close-contact requirement for the action of T3SS and toxin translocation (45) with or without a need for translocation machinery. Using a cell-based screen for additional bacterial factors that may promote ExlA pore-forming activity, we identified *P. aeruginosa* pili as being absolutely required for cytolysis. Bacterial T4P are long extracellular appendages with multiple functions, including bacterial motility and adhesion (38). The role of pili in *P. aeruginosa* T3SS-dependent cell intoxication has been clearly documented in an *in vivo* corneal infection model (46) and in *ex vivo* models of epithelial cells (46–49). They are also required for the injection of T3S toxins; however, the T4P could be exchanged for the nonfimbrial adhesin pH 6 antigen of *Yersinia pestis*, indicating that simple adhesion mediated by pili to cells plays a crucial role in T3S intoxication, presumably by positioning the injectisome onto the host cell surface, whereas the other pilus function, twitching, seems to be irrelevant (50). Cooperation between TPS adhesins and surface appendices involved in adhesion/motility has been previously demonstrated. In *Bordetella*, the fimbriae play a role in promoting further the adhesion properties of the TPS hemagglutinin to eukaryotic cells (51) and TPS protein EtpA binds to the flagella and facilitates *Escherichia coli* adhesion to eukaryotic cells (8). In the case of ExlA, T4P are clearly required for ExlA targeting to the eukaryotic cell membranes, as all pilus mutants secrete ExlA. This indicates that T4P play a role in bringing bacteria into close proximity with their target cell and thus possibly promoting a local increase of ExlA concentration (Fig. 6), a role played by specific lipid and/or protein receptors for other PFTs.

In conclusion, some *P. aeruginosa* strains, lacking T3SSs, have evolved a novel strategy of pathogenesis by acquiring the pore-forming TPS toxin exolysin and using pili as extracellular appendages to facilitate their cytolytic action. Whether this cooperation between the TPS pore-forming toxin and adhesive pili is unique for *P. aeruginosa* or is a conserved mechanism in other bacterial species possessing ExlA-like proteins remains to be investigated.

## MATERIALS AND METHODS

### Bacterial strains, plasmids, and growth conditions.

Bacterial strains and plasmids used in this study are listed in Table S2 in the supplemental material. Bacteria were grown in LB medium supplemented with antibiotics when needed. During construction of deletion mutants, introduction of complementing plasmids, and transposon mutagenesis, the selection for *P. aeruginosa* following mating with *E. coli* was done on LB medium supplemented with Irgasan (25 µg/ml) and antibiotics (75 µg/ml gentamicin [Gm], 75 µg/ml tetracycline [Tc], and 500 µg/ml carbenicillin [Cb]).

10.1128/mBio.02250-16.9 TABLE S2  List of bacterial strains and plasmids. Download TABLE S2, DOCX file, 0.04 MB. Copyright © 2017 Basso et al. 2017 Basso et al. This content is distributed under the terms of the Creative Commons Attribution 4.0 International license.

### Genetic manipulations.

All deletion mutants were obtained by gene splice extension overlap (SOE) PCRs. The *exlA*_*RGA*_ gene was synthesized by GenScript and cloned in BglII-AvrII sites of pUC57. *exlB-exlA*_*RGA*_ was cloned into pSW196 between the EcoRI and SacI restriction sites, to obtain the plasmid *pSW196exlB-exlA*_*RGA*_. For complementation of mutants, wild-type genes were amplified by PCR and cloned into the integrative plasmid pSW196 containing the arabinose-inducible promoter pBAD (52). The constructs in the pSW196 plasmid were transferred into *P. aeruginosa* strains by triparental mating. Primers used in PCR are listed in Table S3.

10.1128/mBio.02250-16.10 TABLE S3  Primers used in this study. Download TABLE S3, DOCX file, 0.02 MB. Copyright © 2017 Basso et al. 2017 Basso et al. This content is distributed under the terms of the Creative Commons Attribution 4.0 International license.

### Cell culture and cytotoxicity assays.

The epithelial cell line A549 (ATCC CCL-185) was grown in 1× RPMI (Gibco Life Technologies) supplemented with 10% fetal bovine serum (FBS; Sigma) at 37°C and 5% CO_2_. Cells were plated in 96-well plates (50,000 cells/well) and incubated for 1 night. Two hours before infection, RPMI medium was replaced by endothelial growth basal medium (EBM-2; Lonza Clonetics). The infection was done at a multiplicity of infection (MOI) of 10 (bacteria per eukaryotic cell). The level of cytotoxicity was determined by measuring the release of lactate dehydrogenase (LDH) using a cytotoxicity detection kit (Roche). Infection supernatants were sampled at 4 h postinfection. Negative controls were noninfected cells; positive controls were cells lysed by the addition of 200 µl of 2% Triton X-100. The optical density (OD) was measured at 492 nm. The extent of cytotoxicity was calculated by determining the percent cytotoxicity.

### Time-lapse microscopy.

A549 cells were plated in 96-well plates (50,000 cells/well) in RPMI medium (1×) for a night. Before infection, the RPMI medium was replaced with EBM-2 and the cells were labeled with vital Hoechst stain (1 μg/ml) and YoPro-1 (1 μM) followed by infection with the bacteria at an MOI of 10. Cell acquisitions were made using the ArrayScan high-content system (Thermo Fisher) every 5 min. Analyses were performed by measuring the fluorescence intensity of each cell using the HCS studio analysis software. Slopes were calculated between the 5 last time points before a sharp increase of fluorescence. The box plot was made using SigmaPlot software (Systat Software Inc., San Jose, CA), and statistical analysis was done by the Kruskal-Wallis test.

For confocal spinning-disk microscopy, the A549-EGFP cells were grown in RPMI medium supplemented with 10% fetal calf serum (all from Lonza). Cells were seeded at 25,000 cells per well on Lab-Tek II 8-chambered (Dutscher Scientific) coverslips and used 24 h later. Cell membranes were labeled after incubation with WGA-Alexa 647 (Life Technologies) for 10 min at 37°C and then infected with bacteria at an MOI of 10 in EBM-2. Cell infections were observed by confocal spinning-disk microscopy for 4 h, with images recorded every 20 min. Successive planes in 3D stacks were taken every 0.5 μm. The 3D reconstruction was performed using the 3D viewer plug-in of ImageJ software.

### Analysis of ExlA secretion.

Secretion of ExlA was monitored either in liquid LB cultures or under infection conditions. A 30-ml bacterial culture, in LB at an optical density at 600 nm (OD_600_) of 1, was centrifuged at 6,000 rpm for 15 min. The supernatants (9 ml) were filtered, followed by addition of 90 µl of 2% sodium deoxycholate (DOC). After a 30-min incubation at 4°C, 900 µl of 100% trichloroacetic acid (TCA) was added and the proteins were allowed to precipitate overnight at 4°C followed by centrifugation at 15,000 × *g* for 15 min at 4°C. Pellets were resuspended in 100 µl of Laemmli loading buffer. For the analysis of proteins during infection of cultured cells, the bacteria were added to 100-mm petri dishes (at an MOI of 10) for 2 h, the supernatants were centrifuged, and the proteins were precipitated with TCA as described above. Samples were separated by SDS-PAGE (12%) and transferred onto polyvinylidene difluoride (PVDF) membranes for Western immunoblotting. Primary rabbit polyclonal antibodies were raised against either synthetic peptides of ExlA (6) or the recombinant C-terminal portion of the ExlA fragment, used at a 1:1,000 dilution. Anti-RpoA (Neoclone) and anti-FliC (39) were used at 1:2,000 and 1:1,000 dilutions, respectively. For the detection of the abundant RpoA protein, the bacterial lysates were further diluted 5-fold. Secondary antibodies were anti-rabbit-horseradish peroxidase (HRP) and anti-mouse-HRP (Sigma). The membranes were developed with Luminata Classico Western HRP (Millipore) substrate.

### Hemolysis assay.

Human red blood cells (RBCs) were obtained from the Etablissement Français du Sang (EFS), Grenoble, France. RBCs were counted, and 5 × 10^7^ were incubated in 100 µl of EBM-2 with bacteria at the MOI of 10 or with 100 nM α-hemolysin from *S. aureus*, in 96-well plates. After centrifugation at 2,000 rpm and gentle agitation, the plates were incubated at 37°C for 90 min. The plates were centrifuged at 2,000 rpm at 4°C, allowing sampling of 100 µl of the supernatant. The optical density was measured at 560 nm. The negative controls were noninfected RBCs, and the positive control was obtained by inducing complete lysis by adding 100 µl of 1% SDS. For osmoprotection assays, RBCs were incubated with 30 mM osmoprotectants in EBM-2.

### Expression and purification of ExlA variants.

ExlA genes were cloned in pET15b for Cter_ExlA_ and in pET28a for ExlA_noSP_ and ExlA_ΔCter_. *E. coli* BL21(DE3) cells were used for the production of ExlA variants. Precultures were done in 30 ml of LB with ampicillin (Amp; 100 μg/ml) for pET15b and kanamycin (Km; 30 μg/ml) for pET28a at 25°C overnight. Expression of the recombinant proteins was induced in a 1-liter culture grown at 37°C by addition of 0.5 mM isopropyl-β-d-thiogalactopyranoside (IPTG) for 1.5 h. Bacteria were centrifuged, and the pellets were resuspended in 20 ml of IMAC10 buffer (25 mM Tris-HCl, 500 mM NaCl, 10 mM imidazole, pH 8) and lysed using a Microfluidizer M_110P2 (Microfluidics, Westwood, MA) at 15,000 lb/in^2^ for 3 min. Soluble fractions were sampled after centrifugation at 30,000 rpm for 30 min at 4°C. Lysates were passed through a Ni^2+^ affinity column (HisTrap HP; GE Healthcare) using the Äkta purifier 10 instrument (GE Healthcare), and the bound proteins were eluted using a 20 mM to 200 mM step gradient of imidazole. Fractions were monitored by gel electrophoresis, pooled, and further purified on cation exchanger (HiTrap SP) and anion exchanger (HiTrap Q) columns (HiTrap; GE Healthcare). Protein concentrations in each fraction were quantified by bicinchoninic acid assay (BCA) using the Optima kit.

### Liposome leakage assay.

L-R-phosphatidylcholine (PC), L-R-phosphatidylethanolamine (PE), L-R-phosphatidylserine (PS), and cholesterol (Ch) were from Avanti Polar. Lipids and liposomes were stored under nitrogen in chloroform at −20°C. Liposomes containing PS/PC (18%/82%), PC (100%), PC/PE (82%/18%), PC/PS/PE (72%/18%/10%), and PC/Ch (80%/20%) were prepared by evaporation of lipids in a rotatory evaporator, hydrated, and resuspended in 1 ml of 25 mM Tris-HCl, 250 mM NaCl, pH 8, containing 50 mM sulforhodamine B by six cycles of freezing-thawing. To obtain large unilamellar vesicles (LUVs) of 100 nm, liposomes were filtered through a 0.1-µm filter by extrusion and the nonencapsulated dye was removed with size exclusion chromatography on a PD10 column (Amersham) equilibrated with 25 mM Tris-HCl, 250 mM NaCl, pH 8. LUV disruption was monitored by an increase of fluorescence intensity upon dye dilution in the same buffer. Measurements were performed on a Jasco FP6500 fluorimeter with excitation set at 565 nm and emission at 586 nm in 2 ml of 10 µM LUV and recorded during 600 s after the addition of the protein. A 10 μM concentration of LUV was incubated with 10 nM protein in Tris or acetate buffer at different pHs. Fluorescence intensities were normalized considering the initial intensity before protein addition and the maximal intensity generated by the addition of Triton X-100 at the end of each kinetics measurement. The initial rates of dye release were derived from the first 10 s of the kinetics, using linear regression.

### Construction of transposon library.

A transposon library was constructed in *P. aeruginosa* IHMA by mating with an *E. coli* donor carrying plasmid pBTK24 containing the *mariner* transposon with a Gm resistance cassette (53). *P. aeruginosa* IHMA was grown in LB at 42°C, and *E. coli* SM10λpir harboring pBTK24 was grown at 37°C with agitation for 18 h. Bacteria in 300 separate aliquots (100 μl) from each culture were combined, concentrated by centrifugation to 50 μl, and spotted on LB agar plates which were further incubated for 5 h at 37°C. Spots were scraped off, resuspended in 100 μl LB, and plated on LB agar plates containing Irgasan (25 mg/ml) and Gm (75 µg/ml) to isolate single colonies for testing in the cytotoxicity assay. The locations of the transposon insertions in candidate mutants were determined by semirandom PCR (primers are listed in Table S2) and sequencing.

### Cellular screen for noncytotoxic mutants by high-content microscopy.

A549 cells were plated in 384-well plates (2,500 cells/well) in 1× RPMI with 10% FBS and incubated overnight. IHMA_tn single colonies from the transposon library were robotically transferred one by one in 96-well plates containing LB with 15 μg/ml Gm. The colonies were then transferred with 96-pin replicators into 384-well plates containing 30 µl of LB and grown statically for 18 h. The day of the screen, A549 cells were washed once with EBM-2, and EBM-2 containing vital Hoechst (1 μg/ml) and Draq7 (3 μM final concentration) stains was added. Infection of cells was initiated with a 384-pin replicator and run for 3 h at 37°C, and then the cells were fixed by adding an equal volume of 2% formaldehyde in phosphate-buffered saline (PBS) to each well. The image acquisition was done using a high-content microscope (ImageXpress Micro; Molecular Devices) at the ICCB Longwood facility, Harvard Medical School, Boston, MA. Analysis was done using MetaXpress software.

### Twitching motility assay.

A determination of twitching motility by *P. aeruginosa* was carried out by inoculating bacteria at the interface between the plastic petri dish and LB agar (10 g/liter tryptone, 5 g/liter yeast extract, 10 g/liter NaCl, 1% agar). After 48 h of incubation at 37°C, agar was removed and the zone of twitching was revealed following Coomassie blue staining.

### Adhesion assay.

A549 cells (5 × 10^5^) were cultured in 1× RPMI with 10% FBS. Before the adhesion assay, A549 cells were treated with trypsin (0.05% EDTA) for 2 min at 37°C, suspended in 1× RPMI with 10% FBS, centrifuged at 2,000 rpm for 5 min, and suspended in EBM-2. Bacterial strains (5 × 10^6^ bacteria) were incubated with cells (MOI of 10) at 37°C with agitation. After 30 min, A549 cells and bacteria were centrifuged at 1,200 rpm for 5 min and pellets were resuspended in EBM-2. CFU in the supernatant and pellet fractions were quantified following serial dilutions in 1× PBS and plating on LB agar.

10.1128/mBio.02250-16.2 TEXT S1 Supplemental materials and methods. Download TEXT S1, DOCX file, 0.01 MB. Copyright © 2017 Basso et al. 2017 Basso et al. This content is distributed under the terms of the Creative Commons Attribution 4.0 International license.
