# Supplementary material for: Pseudomonas aeruginosa Pore-Forming Exolysin and Type IV Pili Cooperate To Induce Host Cell Lysis
Source: mBio. 2017 Jan 24;8(1):e02250-16. doi: 10.1128/mBio.02250-16 (PMC5263249; doi:10.1128/mBio.02250-16)
Supplement: TABLE S1 [file mbo002173153st1.docx]

**Table S1. List of 20 Tn mutants selected after secondary screen as displaying the cytotoxicity of less than 30% compared to the parental strain IHMA.**

| **Gene disrupted or intergenic region^1^** | **Predicted operon** | | **Protein(s)** | |
| --- | --- | --- | --- | --- |
| *pilQ* | *PSPA7_5774 /aroB/ aroK / pilQ/ pilP/ pilO/ pilN/ pilM* | | OM secretin type IV pili | |
| *pilQ* |  |  |  |  |
| *pilW* | *fimU/ pilV/ pilW/ pilX/ pilY1/ pilY2/ pilE* | | minor pilin | |
|  |  | |  | |
| *PSPA7_1554* | *PSPA7_1549 /PSPA7_1550 /PSPA7_1551 / PSPA7_1552 /PSPA7_1553 / PSPA7_1554* | | putative porin | |
| *PSPA7_0652* |  | | hydroxypyruvate isomerase | |
| *PSPA7_6248* | *PSPA7_6247, 49, 50,51* | | FAD linked oxidase | |
| *pqqE* | *PSPA7_3304 / pqqE / pqqD / pqqC / pqqB/ pqqA* | | pyrroloquinolonine quinone biosynthetic protein | |
| *pqqE* |  |  |  |  |
| *mfd* | PSPA7_2156 | | Transcription repair coupling factor | |
| *mfd* |  |  |  |  |
| *mfd* |  |  |  |  |
| *PSPA7_2595* | *ftsK/ lolA/ PSPA7_2595* | | Recombinaison factor protein RarA | |
| *PSPA7_4936 -SOD* |  | | Putative transporter and SOD | |
| *PSPA7_6278 - PSPA7_6279* | |  | | Hypothetical protein |
| *PSPA7_5266 – PSPA7_5267* | |  | | Putative oxidoreductase – hypothetical protein |
| *PSPA7_5735 - PSPA7_5736* | |  | | Glycosyl transferase family protein |
| *PSPA7_2206-PSPA7_2207* | |  | | Putative lipoprotein and hypothetical cytoplasmique protein |
| *PSPA7_4573* | |  | | Hypothetical protein |
| *MO62_20290^2^* | |  | | hypothetical protein, RHS toxin domain |

^1^ Tn junctions were PCR amplified and blasted against the PA7 genome on *Pseudomonas* genome data base (<http://www.pseudomonas.com/>). ^2^ *MO62_20290* is present in the strain RP73. Predicted operons and proteins names are retrieved from *Pseudomonas* genome database.
