# Supplementary figures and images for: Pseudomonas aeruginosa Pore-Forming Exolysin and Type IV Pili Cooperate To Induce Host Cell Lysis
Source: mBio. 2017 Jan 24;8(1):e02250-16. doi: 10.1128/mBio.02250-16 (PMC5263249; doi:10.1128/mBio.02250-16)

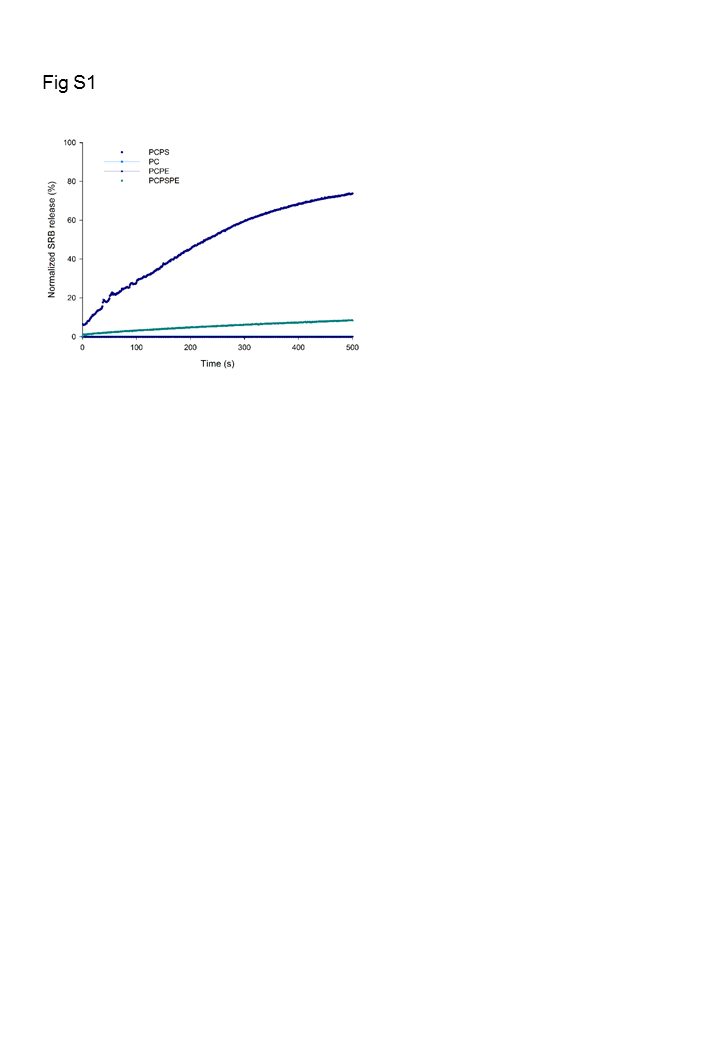

Supplement: FIG S1 [file mbo002173153sf1.tif]

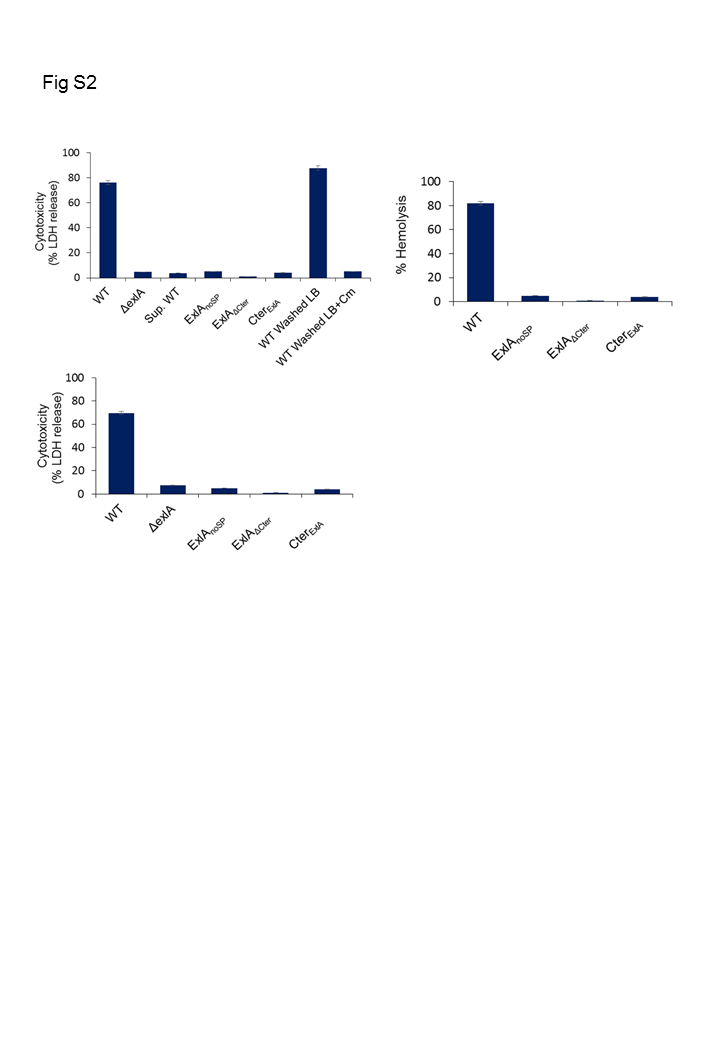

Supplement: FIG S2 [file mbo002173153sf2.tif]

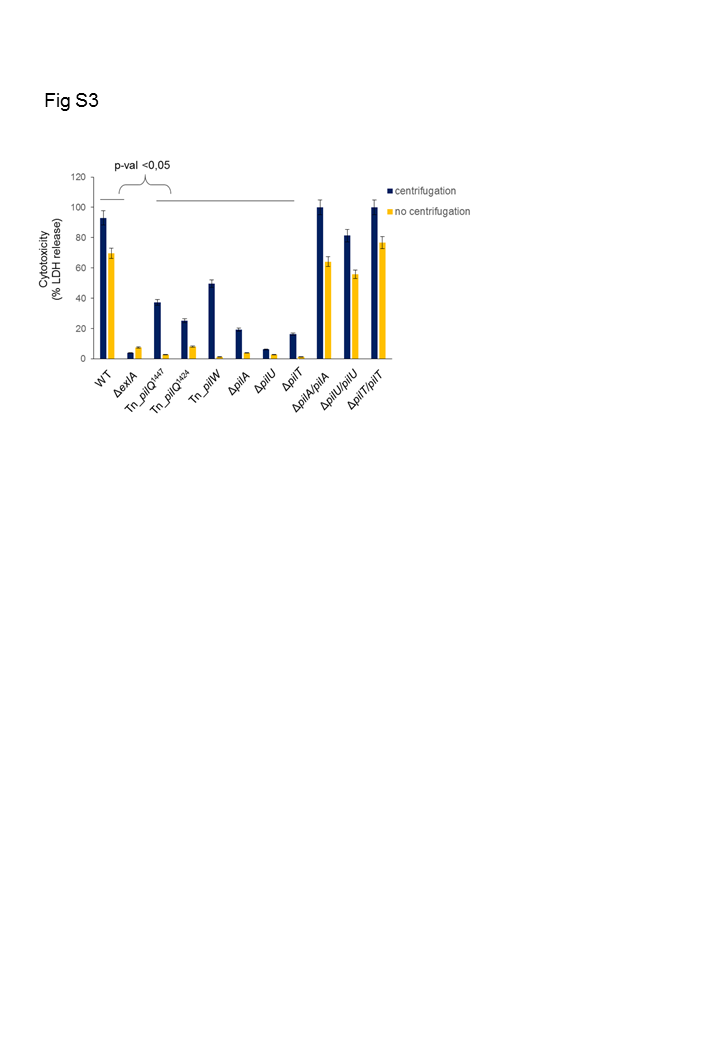

Supplement: FIG S3 [file mbo002173153sf3.tif]

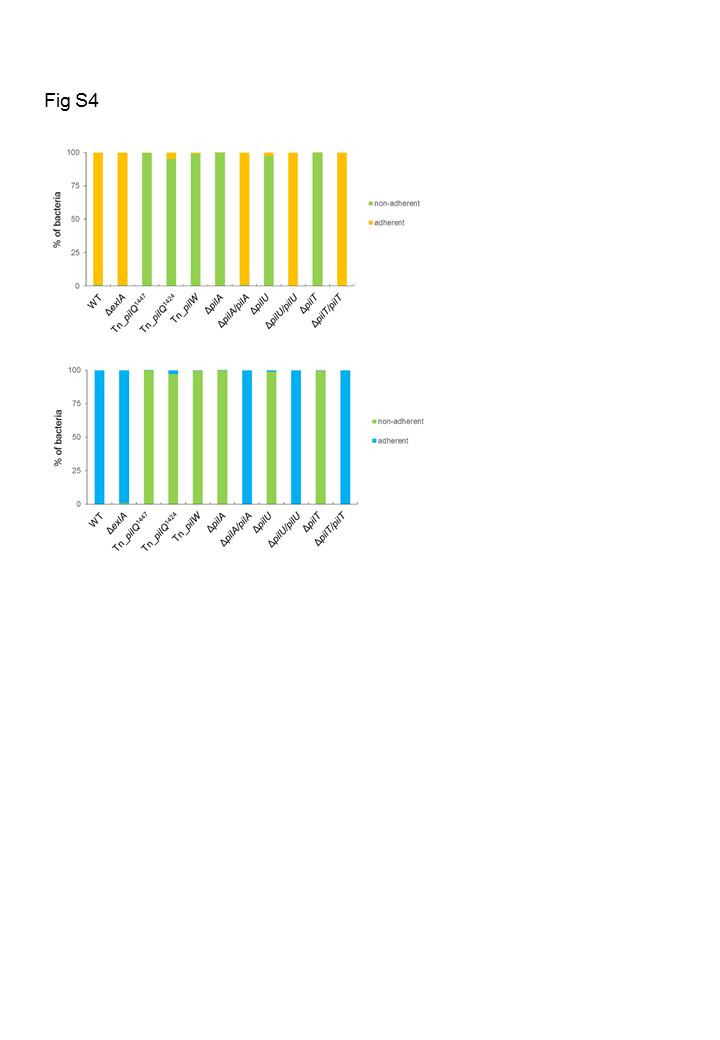

Supplement: FIG S4 [file mbo002173153sf4.tif]

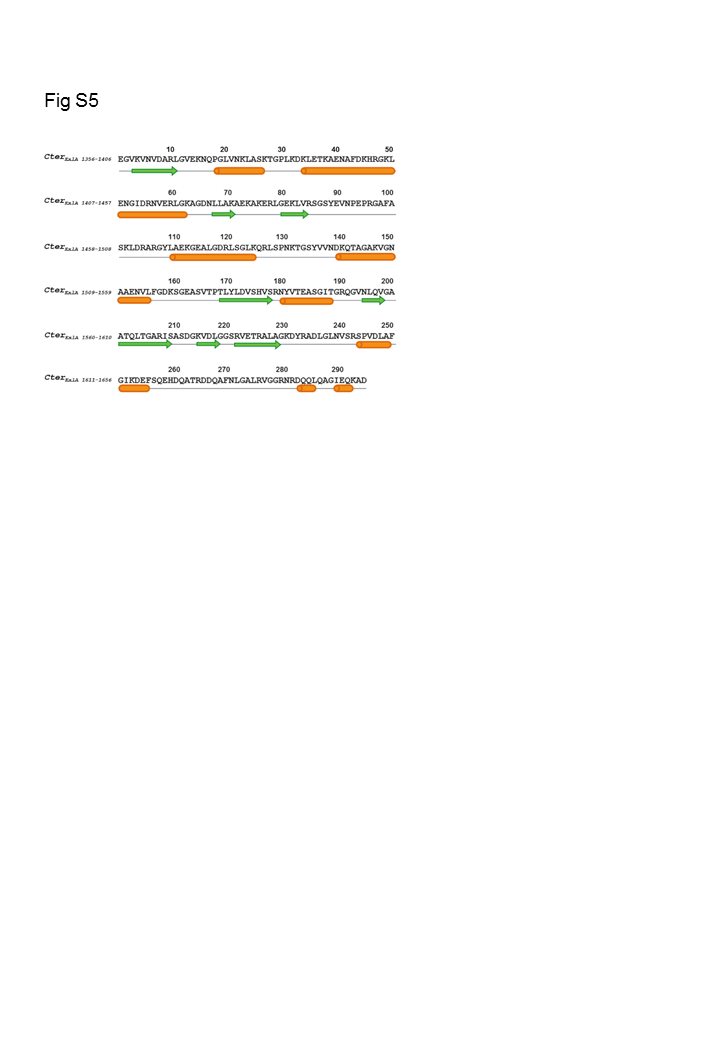

Supplement: FIG S5 [file mbo002173153sf5.tif]
