# Supplementary material for: Pseudomonas aeruginosa Pore-Forming Exolysin and Type IV Pili Cooperate To Induce Host Cell Lysis
Source: mBio. 2017 Jan 24;8(1):e02250-16. doi: 10.1128/mBio.02250-16 (PMC5263249; doi:10.1128/mBio.02250-16)
Supplement: TABLE S2 [file mbo002173153st2.docx]

**Table** **S2. List of bacterial strains and plasmids used in this work.**

| Strains or plasmids | Characteristics | References |
| --- | --- | --- |
| Strains  *Pseudomonas aeruginosa* | | |
| PAO1 Δ*xcpR*Δ*pscC* | PAO1 lacking the Xcp T2SS and T3SS | This work |
| PAO1Δ*xcpR*Δ*pscC::exlBA* | PAO1 mutant with pSW196::*exlBA* | [[1](#_ENREF_1)],This work |
| PAO1Δ*xcpR*Δ*pscC::exlBA_RGA_* | PAO1 mutant with pSW196::*exlBA_RGA_* | This work |
| PAO1Δ*xcpR*Δ*pscC::exlBA_ΔCter_* | PAO1 mutant with pSW196::*exlBA****_ΔCter_*** | This work |
| PAO1Δ*xcpR*Δ*pscC::exlA* | PAO1 mutant with pSW196::*exlA* | [[1](#_ENREF_1)],This work |
| PAO1Δ*xcpR*Δ*pscC::exlB_ΔP1_A* | PAO1 mutant with pSW196::*exlB****_ΔP1_****A* | This work |
| PAO1Δ*xcpR*Δ*pscC::exlB_ΔP2_A* | PAO1 mutant with pSW196::*exlB****_ΔP2_****A* | This work |
| PP34 | *P. aeruginosa* expressing ExoU | [[2](#_ENREF_2)] |
| PP34ΔExoU | Isogenic ExoU mutant of PP34 | [[2](#_ENREF_2)] |
| IHMA879472 (IHMA)* | ExlA+, Urinary strain IHMA | [[3](#_ENREF_3)],This work |
| IHMAΔ*exlA* | *exlA* deletion | This work |
| IHMAΔe*xlA*::*exlBA* | *exlBA* genes cloned into pSW196 and introduced into Δ*exlA* | This work |
| IHMAΔ*pilA* | *pilA* deletion | This work |
| IHMAΔ*pilA*::*pilA* | *pilA* cloned into pSW196 and introduced into Δ*pilA* | This work |
| IHMAΔ*pilT* | *pilT* deletion | This work |
| IHMAΔ*pilT*::*pilT* | *pilT* cloned into pSW196 and introduced into Δ*pilT* | This work |
| IHMAΔ*pilU* | *pilU* deletion | This work |
| IHMAΔ*pilU*::*pilU* | pilU cloned into pSW196 and introduced into Δ*pilU* | This work |
| IHMA879472*pilQ^1447^*_tn | Tn insertion in *pilQ* at nucleotide 1447 | This work |
| IHMA879472*pilQ^1424^*_tn | Tn insertion in *pilQ* at nucleotide 1424 | This work |
| IHMA879472*pilW^127^*_tn | Tn insertion in *pilW* at nucleotide 127 | This work |
| *Escherichia coli* | | |
| DH5α | Cloning strain | Lab collection |
| BL21(DE3) | Protein expression | NEB |
| SM10 ʎpir | Strain used in bi-parental mating | Lory lab collection |
| *Plasmids* | | |
| pSW196 | Tc^R^ , Integrative plasmid at the *attB* site, derived from miniCTX, p*BAD* | [[4](#_ENREF_4)] |
|  |  |  |
| pSW196*exlBA* | pSW196 with *Eco*RI-*Sac*I insertion of operon *exlBA* under p*BAD* | [[1](#_ENREF_1)] |
| pSW196*exlA* | pSW196 with *Eco*RI-*Sac*I insertion of *exlA* gene under p*BAD* | [[1](#_ENREF_1)] |
| pSW196*exlB∆P1-exlA* | pSW196*exlBA* with deletion of POTRA domain 1 of *exlB* | This work |
| pSW196*exlB∆P2-exlA* | pSW196*exlBA* with deletion of POTRA domain 2 of *exlB* | This work |
| pSW196*exlB-exlA_RGA_* | pSW196*exlBA* with penta RGA mutant in *exlA* | This work |
| pSW196exlB-*exlA_∆C-Ter_* | pSW19 *exlBA* with deletion of C-terminal domain of *exlA* | This work |
| pEXG2*pilA* | Gm^R^, suicide plasmid used into *pilA* mutagenesis |  |
| pEXG2*pilT* | Gm^R^, suicide plasmid used into *pilT* mutagenesis | This work |
| pEXG2*pilU* | Gm^R^, suicide plasmid used into *pilU* mutagenesis | This work |
| pSW196*pilA* | pSW196 with *EcoRI-SacI* insertion of *pilA* gene | This work |
| pSW196*pilT* | pSW196 with *Pst*I-*Spe*I insertion of *pilT* gene | This work |
| pSW196*pilU* | pSW196 with *Pst*I-*Spe*I insertion of *pilT* gene | This work |
| pBTK24 | Gm^R^, Plasmid with mariner transposon | [[5](#_ENREF_5)] |
| pRK2013 | Km^R^, helper plasmid | [[6](#_ENREF_6)] |
| pET15b*Cter-ExlA* | Amp^R^ , pET15b with *Nco*I*-Hin*dIII insertion of the Cter domain of ExlA; plasmid used in protein expression | This work |
| pET28a*ExlAnoSP* | Km^R^ , pET28a with *Nde*I*-Hin*dIII insertion of the ExlA^noSP^; plasmid used in protein expression | This work |
| pET28a*ExlAΔCter* | Km^R^ , pET28a with *Nde*I*-Hin*dIII insertion of the ExlAΔCter; plasmid used in protein expression | This work |

Cb^R^: carbenicillin resistance, Tc^R^: Tetracycline resistance, Amp^R^: Ampicilin resistance, Km^R^: Kanamycin resitance

*IHMA: International Health Management Association

**Supplemental References**

1. Elsen S, Huber P, Bouillot S, Coute Y, Fournier P, et al. (2014) A type III secretion negative clinical strain of Pseudomonas aeruginosa employs a two-partner secreted exolysin to induce hemorrhagic pneumonia. Cell Host Microbe 15: 164-176.

2. Berthelot P, Attree I, Plesiat P, Chabert J, de Bentzmann S, et al. (2003) Genotypic and phenotypic analysis of type III secretion system in a cohort of *Pseudomonas aeruginosa* bacteremia isolates: evidence for a possible association between O serotypes and exo genes. J Infect Dis 188: 512-518.

3. Kos VN, Déraspe M, McLaughlin RE, Whiteaker JD, Roy PH, et al. (2015) The Resistome of Pseudomonas aeruginosa in Relationship to Phenotypic Susceptibility. Antimicrobial Agents and Chemotherapy 59: 427-436.

4. Baynham PJ, Ramsey DM, Gvozdyev BV, Cordonnier EM, Wozniak DJ (2006) The *Pseudomonas aeruginosa* ribbon-helix-helix DNA-binding protein AlgZ (AmrZ) controls twitching motility and biogenesis of type IV pili. Journal of Bacteriology 188: 132-140.

5. Kulasekara HD, Ventre I, Kulasekara BR, Lazdunski A, Filloux A, et al. (2005) A novel two-component system controls the expression of Pseudomonas aeruginosa fimbrial cup genes. Molecular Microbiology 55: 368-380.

6. Figurski DH, Helinski DR (1979) Replication of an origin-containing derivative of plasmid RK2 dependent on a plasmid function provided in trans. Proceedings of the National Academy of Sciences of the United States of America 76: 1648-1652.
