# Supplementary material for: Pseudomonas aeruginosa Pore-Forming Exolysin and Type IV Pili Cooperate To Induce Host Cell Lysis
Source: mBio. 2017 Jan 24;8(1):e02250-16. doi: 10.1128/mBio.02250-16 (PMC5263249; doi:10.1128/mBio.02250-16)
Supplement: TABLE S3 [file mbo002173153st3.docx]

**Table S3. Primers used in this study.**

| Primers | Sequences (5’-3’) |
| --- | --- |
| PA7_4642_2F | GCACCAGCCAGGTGAACG |
| PA7_4642_2R | CTCCGCCGCCCTGGCGTC |
| exlB-*Eco*RI | GAATTCGATACATGAAGGATGC |
| exlB-*Sac*I | GAGCTCTCAGATCTGCAGGCTCAG |
| SOE-ΔPOTRA1 _rev_ | GCCGTCGTGGTCTGCGATGG |
| SOE-ΔPOTRA1 _Forw_ | CCATCGCAGACCACGACGGCTACGTCGAGTCCATC |
| SOE-ΔPOTRA2 _rev_ | GGCGCCTTCGTCGACCAGGATATC |
| SOE-ΔPOTRA2 _Forw_ | GATATCCTGGTCGACGAAGGCGCCGCGCGCTGGAGCC |
| Round-1 RndomPA-1 | GGCCACGCGTCGACTAGTACNNNNNNNNNNCGATG |
| Round-1a | GGCCACGCGTCGACTAGTAGNNNNNNNNNCAGCAG |
| Round-1 pBTK | GAAGCTGTGGTATGGCTGTGCAGG |
| Round-2PA | GGCCACGCGTCGACTAGTAC |
| Round-2 pBTK | CGCACTCCCGTTCTGGATAATGTT |
| IHMA-Mut-pilA-F1 | GGATCCGCCGTCGAGATCGACATCGT |
| IHMA-Mut-pilA-R1 | AGCGAAGCGCGCAACATCCTGATTT |
| IHMA-Mut-pilA-F2 | TCAGGATGTTGCGCTTTAGCGCTCAAAATAGATTGCA |
| IHMA-Mut-pilA-R2 | GAATTCGGTAGATTCCGCCAGCGGAGT |
| IHMA-Comp-pilA-F1 | GAATTCTTGGGTTTGGCATGGATCCTGCTGAA |
| IHMA-Comp-pilA-R1 | GGATCCTTAGGGCTTGCTTGCAGGCGCGTTCG |
| IHMA-Mut-pilT-F1 | CC GAA TTC CTG CAG GTA GTT CTC GCC GAA |
| IHMA-Mut-pilT-R1 | CTC GGT AAT ATC CAT GGG ACT C |
| IHMA-Mut-pilT-F2 | G AGT CCC ATG GAT ATT ACC GAG TGA CAC CTG GTA TTC GGC ACC CTG |
| IHMA-Mut-pilT-R2 | CC CCC GGG CTA GAC GCA GTT CCG GGA TTC |
| IHMA-Comp-pilT-F | CC CTG CAG ACG GCG GCT TTG GCG GC |
| IHMA-Comp-pilT-R | CC ACT AGT CTA GAC GCA GTT CCG GGA TTC |
| IHMA-Mut-pilU-F1 | CC CTG CAG CGA GAA GGC GAA GAT CCC GGA |
| IHMA-Mut-pilU-R1 | TCA CTG CTC GTT CAT CAC GCC GAG |
| IHMA-Mut-pilU-F2 | GGC GTG ATG AAC GAG CAG TGA ATC GAG GTG CTG CTG AAC ACT C |
| IHMA-Mut-pilU-R2 | CC CTC GAG ACT AGT CGC TAC GAG ATC GAA CAC AGC A |
| IHMA-Comp-pilU-F | CC CTG CAG CCA AAT CCT TGT CGG CGA GCG |
