# Supplementary material for: Pseudomonas aeruginosa Pore-Forming Exolysin and Type IV Pili Cooperate To Induce Host Cell Lysis
Source: mBio. 2017 Jan 24;8(1):e02250-16. doi: 10.1128/mBio.02250-16 (PMC5263249; doi:10.1128/mBio.02250-16)
Supplement: TEXT S1 [file mbo002173153s1.docx]

Running title: *P. aeruginosa* Exolysin and T4P

***Pseudomonas aeruginosa* pore-forming Exolysin and Type IV pili**

**cooperate to induce host-cell lysis
Basso et al.**

**Supplemental methods**

**Cell culture and cytotoxicity assays**

RAW cell line was grown in DMEM without pyruvate (Gibco Life Technology) supplemented with 10% Fetal Bovine Serum (FBS, Sigma) at 37°C, 5% CO_2_. Cells were plated in 96-well plates (50,000 cells/well) and incubated for one night. Two hours before infection, DMEM medium was replaced by endothelial growth Basal Medium (EBM-2) (LONZA Clonetics) medium. The infection was done with a Multiplicity of Infection (MOI) of 10. In the conditions with centrifugation, cells after infection were centrifuged at 4,000 rpm for 10 min, 37°C. The level of cytotoxicity was determined by measuring the release of Lactate Dehydrogenase (LDH) using the Cytotoxicity Detection Kit, Roche. Negative controls were non-infected cells; positive controls were cells lysed by the addition of 200 µl of 2% TritonX-100. The optical density (OD) was measured at 492 nm. The percentage of cytotoxicity was calculated with % cytotoxicity= (OD-OD min)/(OD max-OD min)x100.

**Adhesion assay**

RAW macrophages were culture in DMEM 1X with 10 % of FBS. Before the adhesion assay, cells were treated with trypsin (0,05% EDTA) for 2 min at 37°C, suspended in DMEM 1X 10 % FBS, centrifuged at 2,000 rpm for 5 min and suspended in EBM-2 medium. For the condition with cytochalasin D, macrophages were incubated 1 hour before infection with 1µM of cytochalasin D. Bacterial strains were incubated with cells at MOI 10 at 37°C with agitation. After 30 min, macrophages and bacteria were centrifuged at 1,200 rpm for 5 min. Supernatants and pellets were collected and colony forming units (CFU) were quantified by serial dilutions in PBS 1x and plating on LB agar.
